# Conducting online virtual environment experiments with uncompensated, unsupervised samples

**DOI:** 10.1371/journal.pone.0227629

**Published:** 2020-01-30

**Authors:** Bernd Huber, Krzysztof Z. Gajos

**Affiliations:** School of Engineering and Applied Sciences, Harvard University, Cambridge, MA, United States of America; Monash University, AUSTRALIA

## Abstract

Web-based experimentation with uncompensated and unsupervised samples allows for a larger and more diverse sample population, more generalizable results, and faster theory to experiment cycle. Given that participants are unsupervised, it is still unknown whether the data collected in such settings would be of sufficiently high quality to support robust conclusions. Therefore, we investigated the feasibility of conducting such experiments online using virtual environment technologies. We conducted a conceptual replication of two prior experiments that have been conducted in virtual environments. Our results replicate findings previously obtained in conventional laboratory settings. These results hold across different device types of participants (ranging from desktop, through mobile devices to immersive virtual reality headsets), suggesting that experiments can be conducted online with uncompensated samples in virtual environments.

## Introduction

With more and more human subjects research being conducted through online experiments and crowd work, a novel research direction is to investigate uncompensated samples as a way to conduct large scale studies with the benefit of being cheaper and better representative populations [1, 2]. Previous studies have shown that conducting large scale online experiments with unpaid volunteers has little effect on data quality, while providing potentially much more ecologically valid data compared to paid alternatives [3].

Virtual environment technology holds many promises, among which is the potential to enable new methods for conducting social and psychological experiments [4]. With wider adoption of devices and developer platforms supporting virtual environment technologies, such as WebVR [5] or Google Cardboard [6], there is new promise in the kind of studies and experiments researchers can do in order to get insights that may not have been possible to get before. Despite the promise and scale of adoption of such technology, most experiments in virtual environments, with a few notable exceptions (e.g., [7–9]), are conducted in physical lab spaces or paid settings.

In this work, we study voluntary, unpaid participants of online experiments in virtual environments. Such experiments are designed to be intrinsically motivating (users can often learn about themselves) [10]. We replicate two previously studied phenomena. The first study investigates people’s navigation abilities by letting participants escape virtual mazes [11]. In this study, male participants solved a maze significantly faster compared to female participants. The second study investigates the *Proteus Effect*, which predicts that people’s confidence changes when their perceived appearance is being manipulated. Specifically, one of the findings of this study is that perceived participant height influences confidence as expressed by negotiation behavior in the ultimatum game [12, 13].

We designed and deployed two experiments for LabintheWild.org [3], an online experiment platform, based on those two phenomena. Since both original studies were conducted in compensated settings, and we studied participants in an uncompensated setting, we recruited users by offering them feedback about their navigation style, and their negotiation skills, respectively. Our experiments suggest that both experiment approaches can be used to study human behavior at similar quality levels that supervised and compensated settings provide, suggesting the value of studies in virtual environments. In summary, this work contributes the following:

- Replication of virtual environment studies in uncompensated online settings with the redesign of incentive structures for such settings.
- Demonstration of feasibility of embodiment and place illusion as manipulation mechanisms for such experimentation settings.
- Study of the effect of device type in uncompensated settings.

## Background

In our work, we leverage the advantages of experiments with uncompensated samples, as well as behavioral studies in virtual environments. The following section provides an overview of both of those areas, as well as the conceptual background of our work.

### Uncompensated online behavioral experiments

Online experiments have become an acceptable tool in behavioral research [14]. There are many potential benefits for conducting experiments using uncompensated online studies. According to Reinecke and Gajos [3], benefits include (1) subject pool diversity in terms of age, ethnicity, and socioeconomic status; (2) very low cost to running studies; (3) fast theory/experiment cycle; (4) relative stability of the subject pool over time. People on such experiment platforms typically arrive at the experiments through various sources such as referral, news articles, or social media. A major additional advantage is on the potential scale of distribution, depending on the study setup. Together with the negligible costs, online experimentation with uncompensated samples allows for studies with larger numbers of participants with very long duration. The larger scale and diversity provides an opportunity to apply more granular treatments.

Despite the advantages of running studies “in the wild”, there are various challenges to conducting studies out of the lab. These include reliability of data gathering, as well as ensuring the control of conditions. Previous work studies the effect of bringing lab-based findings online, both in compensated and uncompensated settings. [3, 15] The results suggest that while incentive structures may differ between compensated settings and uncompensated settings, collected data does not necessarily suffer in uncompensated settings [3]. Additionally, mechanisms such as a survey question that simply asks participants whether participants cheated while taking a test, has been shown to capture a wide range of noise. In another study, Komarov et al. [15] compare supervised lab-based user studies with unsupervised online studies on Amazon Mechanical Turk, finding that unsupervised settings lead to similar results. Our work extends these findings to experiments in virtual environments.

### Behavioral studies in virtual environments

Virtual reality (VR) has become an important tool for studying behavioral and cognitive processes since Blascovich et al.’s call for using VR as a research tool in 2002 [4]. This section provides a short overview of some areas of experimental work.

#### Large-scale

Internet-based VR studies have recently become more popular. Gehlbach et al. replicated in 2015 an earlier study on perspective taking conducted in VR using Amazon Mechanical Turk (AMT) with a desktop VR [9]. In 2016, Oh et al. proposed the concept of Immersion at Scale, testing out collecting data on mobile VR devices outside of the lab by setting up physical tents at different locations (e.g., at local events, museums) [7]. Researchers also conducted the first ethnographic study in VR with remote participants [16]. More recently, researchers investigated paid crowdsourced VR experiments [8], in which three studies were replicated and the feasibility of using head-mounted VR in crowdsourcing settings was shown. Furthermore, Mottelson et al. [17] find that results quality holds when moving lab-based VR studies to outside-the-lab VR settings, with the complexity of the studied phenomenon governing this effect. Steed et al. replicate test the effect of presence and embodiment in VR “in the wild” by replicating lab studies [18]. We extend previous work on VR studies, by moving towards uncompensated online settings, and including non-head mounted devices in our analyses.

#### Enhancing mediated experiences

An immersive experience can be described as one in which a person is enveloped in a feeling of isolation from the real world [19]. For example, games in three-dimensional environments and with high degrees of interaction often make gamers feel immersed with the virtual environment [19]. A related aspect of a virtual experience is *presence*: the extent to which a person’s cognitive and perceptual systems are tricked into believing they are somewhere other than their physical location [20]. The concept of presence is a frequently emphasized factor in immersive mediated environments. Previous research often assumes that greater levels of immersion elicit higher levels of presence, in turn enhancing the effectiveness of a mediated experience. Cummings et al study the effect of levels of immersion on presence, drawing from multiple studies conducted in VR [21]. Their findings suggest that immersion technologies such as stereoscopic visuals, wider fields of view and increased user tracking have medium sized effects on presence, while other technological factors such as visual content quality have less of an effect on presence. The authors also speculate that these effects may change, as technology becomes more and more adopted.

#### Models of illusion

Gonzales-Franco and Lanier argue that VR is capable of delivering primarily three types of illusions: place illusion, embodiment illusion, and plausibility illusion [22]. Place illusion refers to a user’s feeling of being transported into a rendered environment. Embodiment illusion refers to a user’s feeling of experiencing the virtual world through an avatar. Together, place and embodiment illusions enhance the plausibility illusion, which refers to the feeling that events happening in the virtual world are real. In general, researchers have been leveraging all three types of illusions in their studies to deliver different experimental manipulations. The value of the illusions is that it allows researchers to study phenomena that would have been hard to manipulate or study outside of VR since it is often much harder to manipulate some of these factors in the real world. In this work, we study place and embodiment illusions as mechanisms for such experiments. The following sections describe these factors at greater detail.

Place illusion builds on real-world human behavior where environments interact with how humans behave in a situation. For example, Maani et al. showed that immersion in cooling virtual environments during surgical procedures can reduce perceived pain levels [23]. In another example, researchers put participants in a virtual forest, replicating the behavior of hikers in a previously studied real-world experiment [24].

Embodiment illusion is the experience of a virtual world through a virtual self-representation, often referred to as an avatar. Many studies using VR technologies have demonstrated the influences of embodied experiences on behavior. Research has shown that it is possible in VR to generate perceptual illusions of ownership over a virtual body seen from a first-person perspective, and learn to control the virtual body even when the body appears different from the user’s real body. In addition, different avatar designs have been shown to affect perceived levels of presence and other behaviors. An often studied phenomenon in virtual environments regarding the embodiment illusion is the Proteus effect [13, 25]. The Proteus effect refers to the phenomenon that characteristics of a user’s avatar influence the user’s behavior in a virtual environment. Yee and Bailenson showed for example that participants assigned more attractive avatars behaved more intimately with confederates in self-disclosure and interpersonal distance tasks, and participants assigned taller avatars behaved more confidently in a negotiation task [13, 25]. Additional support of the Proteus effect was exhibited in a study in which the embodiment of sexualized avatars elicited higher reports of self-objectification [26]. While other follow-up studies found opposite effects of less attractive appearance leading to more positive behaviors [25], previous literature agrees on the fact that appearance-related attributes of self-images lead to changes in participant behavior.

## Overview

Given the advantages of online experimentation with uncompensated samples and the promising developments of technology supporting virtual environments, we pose the research question of whether experiments in such settings are feasible. We considered virtual environments in both immersive settings such as HMD, as well as non-immersive environments such as Desktop VR [23]. Specifically, we pose the following questions:

**RQ1** Is it feasible to study the effect of gender on spatial abilities in virtual environment online experiments with uncompensated samples?
**RQ2** Is it feasible to study the effect of appearance on behavior in virtual environment online experiments with uncompensated samples?
**RQ3** How do these effects vary between different devices?
**RQ4** What is the population and knowledge of VR online experimentation participants?

## Study 1: Spatial navigation task

In the first study, we examined people’s navigation abilities with the place illusion. Previous research had shown that in navigation settings without points of interest, gender affects people’s abilities to navigate [11]. In the original study, researchers let participants escape virtual mazes without showing them any map or overview. Participants were using a virtual environment at a desktop computer in a supervised laboratory setting. Gender differences were significant on maze completion times and errors in the maze made by participants. Furthermore, differences in errors by gender varied between trial number: while a significant main effect of gender on error rate was found, no significant effect of gender was found when only looking at the first trial of a maze. The authors conclude from this observation that men and women learn differently, with men learning faster than women. We modeled our first study after this experiment.

### Methods

This work was approved by the Harvard University-Area Committee on the Use of Human Subjects (IRB Registration—IRB00000109; Federal Wide Assurance—FWA00004837), with Protocol#: IRB17-1989. Participants were presented with an informed consent form at the beginning of the study.

#### Tasks and procedures

**Fig 1:**
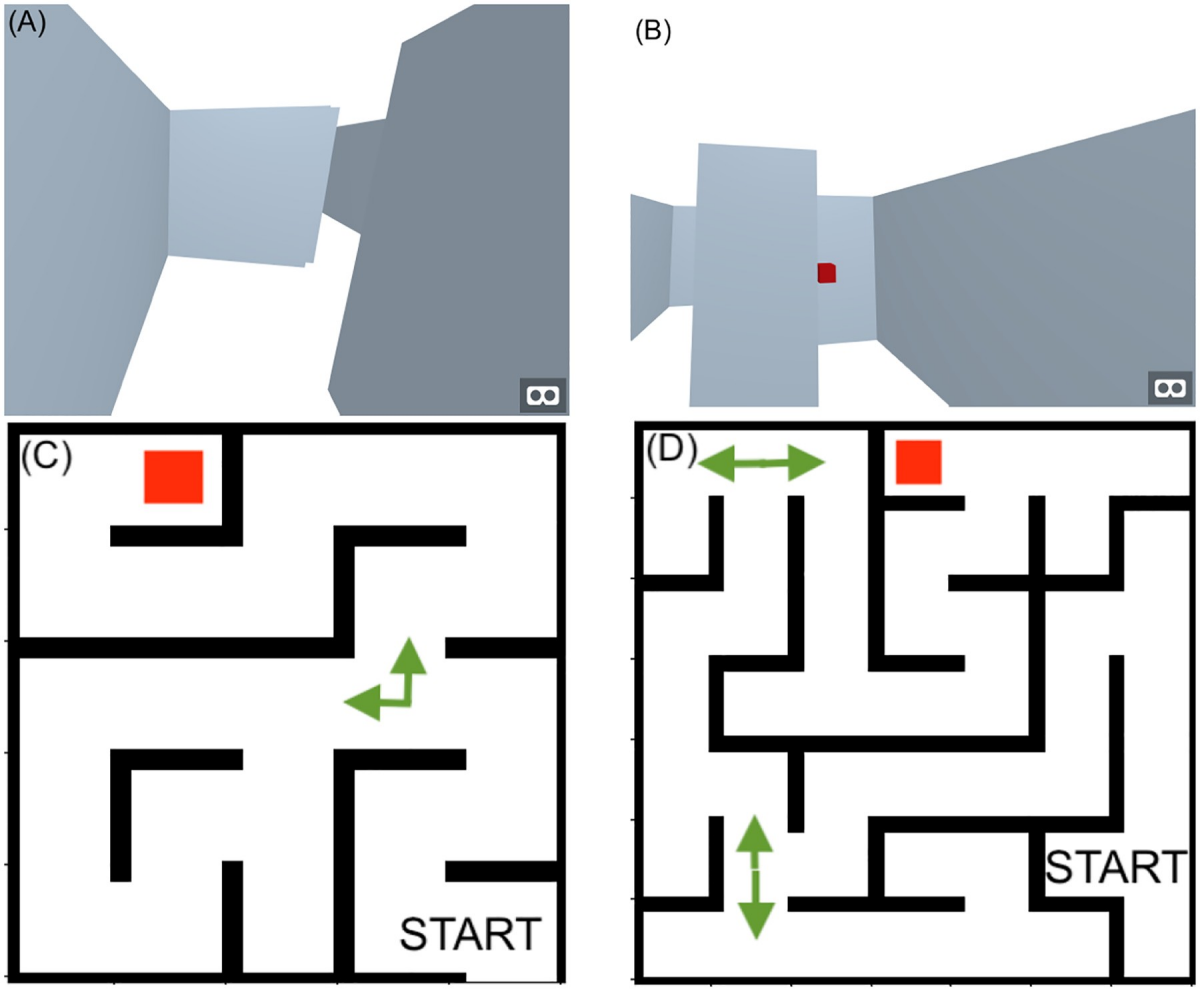
(A, B) Participants’ view within the maze, and the view of the goal (red box). (C,D) The layout of the two mazes. The left, simple maze has one decision point on its path to the goal, the difficult maze has three decision points on its path to the goal. Note that this layout was never shown to participants.

Following the experimental procedure in [11], participants were given multiple trials to escape a maze, without any other information about the map, their current location in the maze or landmarks. Participants were prompted to remember their way out and to complete the task as quickly and accurately as possible. The exit of a maze is marked with a red box. Participants were asked to walk toward the box to finish the maze. See Fig 1 for the mazes we used and the look of the virtual maze environment.

We adapted the original study in several ways. Running the study online in unsupervised settings and without compensation required us to keep the maze experience rather short. Instead of five trials per maze as in the original paper [11], we used three trials per maze. Furthermore, in the original study, multiple decision points were put into every maze. We added one and three decision points per maze, respectively.

We used camera tilt to control forward and backward motion, and the viewer’s orientation to control the moving direction. On a mobile device, the camera tilt can be controlled by the user with the angle of the device. On a desktop device, the camera tilt can be controlled by dragging the camera view with the mouse pointer. We designed the test this way to have the least keyboard interactions necessary, and to have consistent controls across devices.

After giving their informed consent, participants were asked to fill out a demographics questionnaire. They then received instructions about the experiment, and we asked them about the current device they used (Head-mounted, mobile or desktop). Users could then test the motion controls in a tutorial environment without maze. As a next step, participants entered the first (easier) maze and were prompted to find the exit. Participants had three trials for this first maze to optimize their time needed to escape the maze. Participants then entered the second maze (difficult), with the same task to optimize their escape time. Instructions were presented in English.

#### Designing for uncompensated samples

In the original procedure, financial rewards were given to the study participants. In our case, we needed to design the study such that it attracts intrinsically motivated participants, providing non-monetary value. Participant motivation is also important for recruitment in uncompensated settings [27].

**Fig 2:**
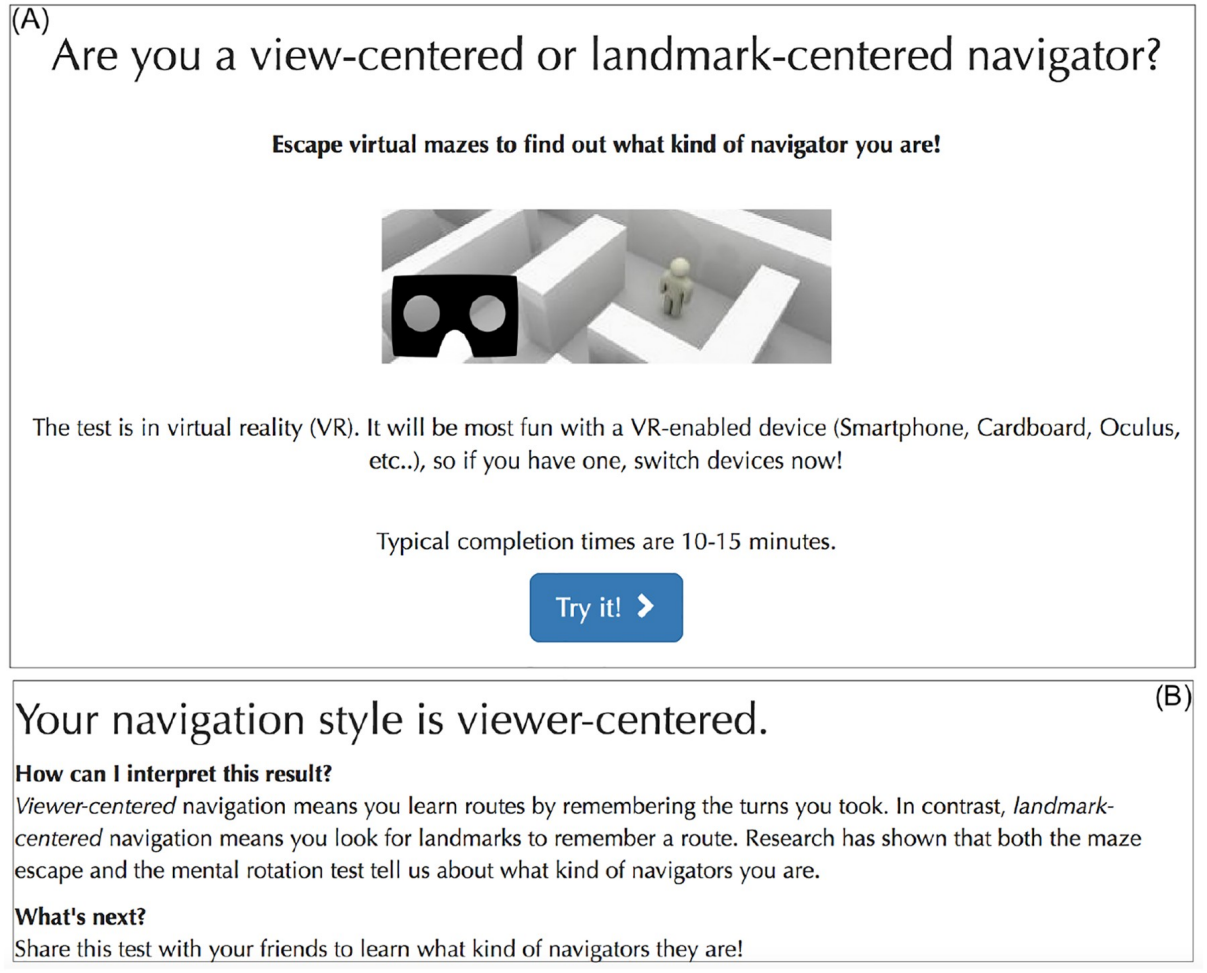
(A) The recruitment page of the navigation study. Participants are prompted to participate in order to learn about their navigation style. (B) The results page of the navigation study. Participants are assessed based on their traveled distance and the time they needed. Participants are also provided additional material explaining the test results in greater detail.

In our case, we provided people an assessment of their navigation style, since the original paper [11] hypothesizes that gender differences in the scores of this navigation test come from the fact that female participants have different navigation styles more centered around landmarks. When participants performed below average, their navigation style was classified as landmark-centered. When participants performed above average, their navigation style was classified as view-centered. See Fig 2 for screenshots of the recruitment and results pages.

#### Participants

311 participants completed all six maze trials. The participants (51% female) were from 52 different countries. Participants were between 12 and 71 years old (mean = 31, sd = 11 years). Participants were also asked how often they used computers, and how often they used HMD devices.

#### Design and analysis

To investigate the possibility of gender differences in time-to-completion and error rates, a repeated measures analysis of variance (ANOVA) was performed with gender as a between-subjects factor and maze difficulty, computer usage (how often the participant uses a computer) and device type (Desktop, Mobile, HMD) as control variables, as well as trial number as a repeated measure. We furthermore included an interaction effect between gender and trial number to measure differences in learning rates between men and women.

Completion times were computed in seconds as the time from when the participants entered the maze, to the moment when they escaped. Error counts were computed as the number of times when participants chose a wrong turn at a decision point. Specifically, every time a participant passed a decision point and chose a wrong turn, the error count was increased by 1, which we adopted from the original study.

### Results

**Fig 3:**
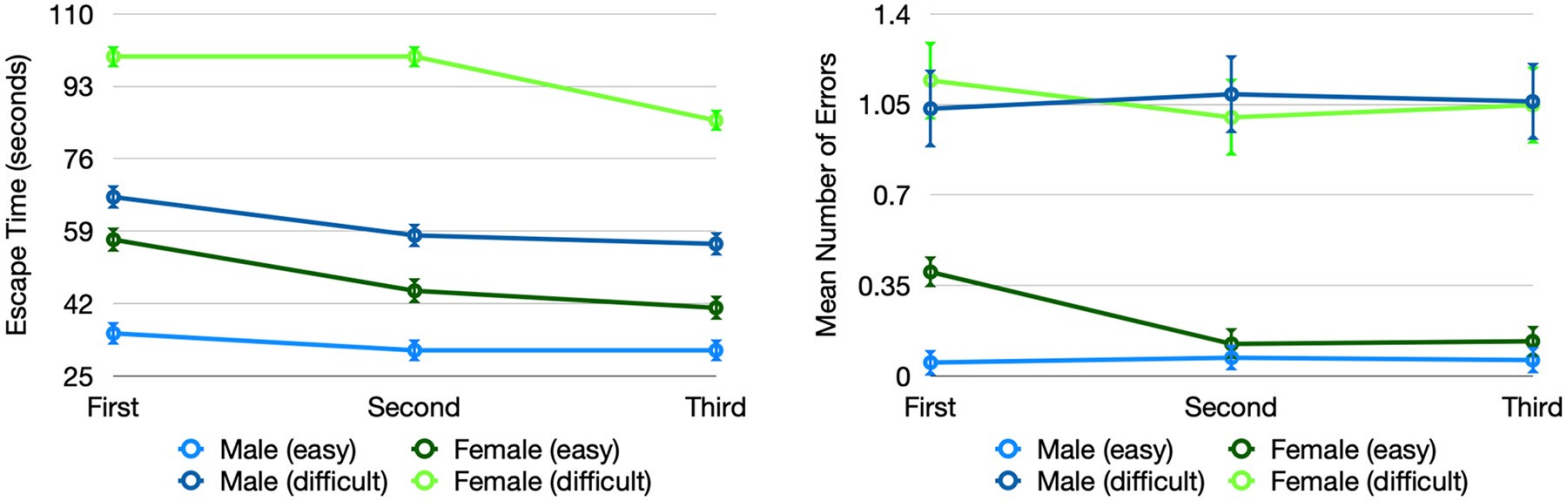
(A) Performance of participants as measured by completion times in seconds, divided by gender and maze difficulty. (B) Errors made by participants at decision points, divided by gender and maze difficulty.

Fig 3 shows the mean times and error rates of male and female subjects to complete the three maze trials for the two different mazes. The overall average escape times were 62 seconds for the simple maze and 78 seconds for the difficult maze, in the last rounds for each maze (see Table 1 for detailed result statistics). Participants traveled 1.2 and 1.4 times the optimal route (averaged over all trials), for the simple and difficult maze respectively.

**Table 1:** Mean and standard deviations (in brackets) of error rate and completion times separated by gender and maze difficulty.

Gender	Maze Difficulty	Number of Errors	Completion time (seconds)
Male	Maze Easy	0.06 (0.16)	30 (18)
	Maze Difficult	1.06 (0.15)	55 (27)
Female	Maze Easy	0.21 (0.28)	48 (28)
	Maze Difficult	1.06 (0.15)	81 (34)

The results of the ANOVA analysis did show a statistically significant main effect of gender on completion time (*F*(1, 518) = 63.2, *p* =< .0001) when controlled for maze difficulty, computer usage of participants, and device type, with male participants solving the maze significantly faster compared to female participants. As well, there were significant main effects of trial number (*F*(2, 517) = 5.0, *p* = 0.0073), with participants getting faster at solving the maze by doing it multiple times (see Fig 3). In contrast to the original study, our data show a significant interaction effect between the trial number and gender (*F*(2, 517) = 9.2, *p* = .0020), with female participants improving their completion times faster (6% average improvement rate between trials) than males (3% average improvement rate between trials). No significant effect of device type (HMD, mobile, desktop) on performance was found (*F*(2, 517) = 3.72, *p* = 0.066). In addition, our data did not show an interaction effect between device and gender.

For error rates, our ANOVA with the same control variables showed a statistically significant main effect of gender (*F*(1, 518) = 35.2, *p* < .0001), with male participants committing significantly fewer errors compared to female participants. Furthermore, there were significant main effects of trial number (*F*(2, 517) = 7.71, *p* = .0005), and device type *F*(2, 517) = 12.3, *p* = 0.0005, with participants on desktop committing significantly fewer errors. Our data also shows a significant interaction effect between trial number and gender (*F*(2, 517) = 6.1, *p* = .0023), with female participants reducing their error rates faster (6% average improvement rate between trials) than males (0.5% average improvement rate between trials). Table 2 summarizes our results and compares them with the results in the original study.

**Table 2:** Summary of our statistical analyses for the maze study, compared to the analysis results reported in the original study.

		Original Study			Ours		
		F	df	p	F	df	p
**Completion Time**	**Gender**	20.3	1,71	<.001	63.2	1, 518	<.0001
	**Trial Number**	14.74	4,288	<.001	5.0	2, 517	.0073
**Error Rates**	**Gender**	17.41	1,71	<.001	35.2	1, 518	<.0001
	**Trial Number**	13.81	4,288	<.001	7.71	2, 517	.0005

### Discussion

**Fig 4:**
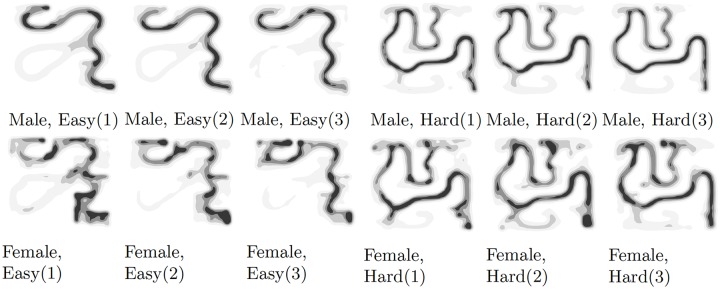
Heatmap of the walking path, divided by gender, maze type (easy, hard), and trial number. The heatmaps visualize participants improvement in escaping the mazes when trying multiple times. Furthermore, the heatmaps show gender differences in walking paths.

We replicated results from the original study for both completion time between gender, as well as error rates. Main effects of gender on maze completion times that we observed are aligned with previous work, suggesting a gender difference on spatial navigation tasks without landmarks. In Fig 4, the path heatmap, as divided by gender, shows that decision points were the most important struggle causing this difference. Furthermore, based on our data analyses, participants were able to learn to navigate the mazes since their completion times became lower with an increasing number of trials per maze. This seems to be also reflected in the heatmap of participants’ walking paths. Our data furthermore reveals that especially as the navigation task gets more difficult (more decision points, more turn points), female participants more likely learn the optimal paths with increasing trials.

One conclusion from the original study was that the difference in error rates between male and female participants can be explained by separating information errors from spatial memory errors. While in the first trial, errors occur due to a lack of knowledge of the correct route (information error), consecutive errors are more likely made due to spatial memory errors. The original study finds an increase in the gap of error rates between male and female participants over the number of trials. Our findings show a different pattern, showing the significant interaction effect between trial number and gender and female participants learning faster than male participants. Our error analysis also revealed that participants often corrected the error at one decision point, while introducing an error at another decision point. It remains an open question what the mechanics are of how learning of the maze impacts performance.

The replication of the results from a lab study in uncompensated online settings shows that it is possible to deliver place illusion in such settings, with the main effect of gender on performance remaining intact. That we were able to replicate the experiment results from laboratory settings and across multiple device categories (RQ3) suggests that such experiments can be used to study people’s navigation behavior in uncompensated settings (RQ1). Even in such settings as uncompensated online experiments, we were able to replicate observations about people’s navigation abilities. While our sample size is still relatively small compared to non-VR experiments in uncompensated online experiment settings, this experiment shows the potential of using virtual environments to conduct immersive studies online without compensation.

## Study 2: Negotiation task

In our second study, we examined the Proteus effect. The Proteus effect refers to the phenomenon that *individual’s behavior conforms to their digital self-representation independent of how others perceive them*. This effect is an often studied topic in VR [13, 25]. The original paper that coined the term showed in one of the experiments that *participants assigned taller avatars behaved more confidently in a negotiation task than participants assigned shorter avatars*. In the original study [13], the authors tested the effect of appearance in a virtual environment on confidence in the negotiation behavior of 50 undergraduate university students, letting them negotiate in a lab-based virtual environment. In the study, height was manipulated relative to the confederate, allowing participants to infer their own height. Negotiation differences were significant between different heights that were randomly assigned to participants. The original study also looked at other appearance-related manipulations such as facial look. Participants were using an HMD in a supervised laboratory setting. In our replication study, we asked whether the same overall methodology can be effective with less immersive (but more pervasive) devices and with unsupervised online participants.

### Methods

#### Tasks and procedures

The Proteus effect study manipulated the height of a user’s avatar in a virtual environment and measured participants’ confidence via the behavior in a negotiation task against another virtual avatar operated synchronously by a confederate. The negotiation implemented was a version of the Ultimatum Game [28], in which a hypothetical pool of $100 was split between the negotiating parties; one party chose a split and the other chose either to accept it (in which case, the money was shared accordingly) or to reject it (nobody received any money). Taller (in the virtual environment) and therefore more confident negotiators were hypothesized to suggest more skewed splits, and more readily reject unfair splits. In our study, we used the same task of the Ultimatum Game to observe the impact of height manipulation on participant behavior.

**Fig 5:**
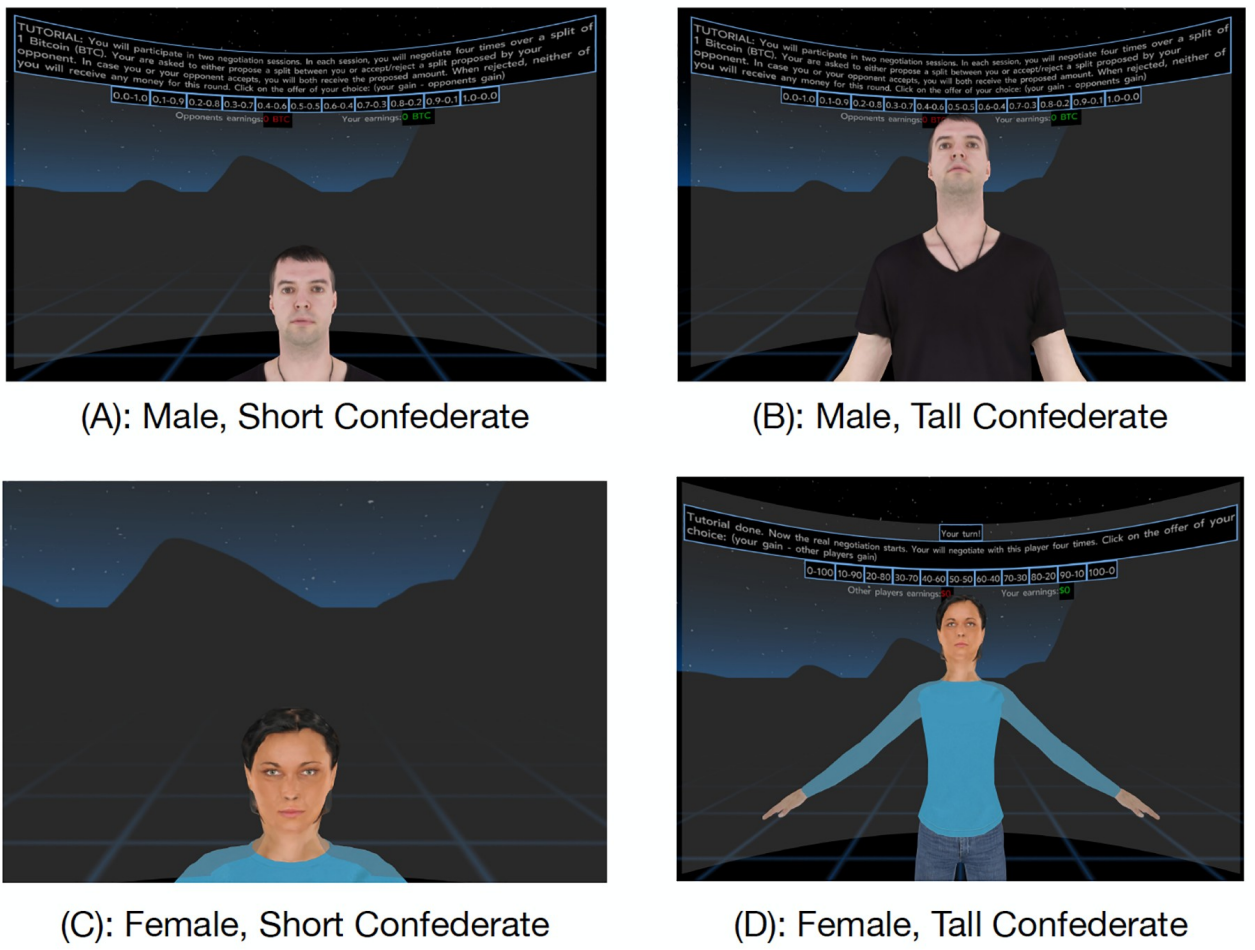
Participants’ view in the conditions *short* and *tall*, in the two different gender conditions. Note that only gender is varied within-subjects, while the height condition is only manipulated between subjects.

We adapted the original study in several ways. Running the study online with uncompensated samples, we had to devise a way to run it without a real confederate, and without monetary reward. We used a virtual confederate that was programmed to make specific bids and accept or reject offers according to consistent guidelines not revealed to participants. We also created a different manipulation of avatar height. Instead of showing both the user avatar and the avatar of the confederate of a different height, the user only saw the confederate avatar, whose scale was manipulated to be smaller or larger as a proxy for participant’s height (see Fig 5). Finally, while the original study had the participants always play against an avatar of the opposite gender, we had each participant play against two avatars in total (one male and one female, in randomized order).

On the recruitment page, participants were shown a brief description of the study. When participants decided to take the test and clicked on a button to enter the test, they were asked to provide their informed consent. Participants were then asked to fill out a short demographic questionnaire. They then received further instructions about the experiment, and we asked them about the current device they used (HMD devices such as Cardboard or Oculus, mobile or desktop). Before the actual task and before seeing the confederate avatars, each participant first went through a tutorial about the Ultimatum Game and was asked to pass two test rounds of the game to make sure they understood the rules. The participant then played one set of a four-round Ultimatum Game with the first opponent, proposing to split in the first and third round. Consistent with [13], the confederate avatar was programmed to always accept a split if the amount proposed to give the avatar is equal or more than $20. The avatar was also programmed to offer 50-50 and 25-75 split in favor of the avatar in the second and fourth round. At the completion of the first set of rounds, the same procedure was repeated for the second opponent for another four rounds. To support realistic play, we told participants they would learn about their negotiation skills depending on how they negotiate and depending on their rank in terms of the total amount of money retained in the game. The test took about 5 minutes to complete.

#### Designing for uncompensated samples

Previous research that studied the Proteus effect mostly used financial rewards to incentivize participation. In our case, we needed to design the study such that it attracts intrinsically motivated participants, providing non-monetary value.

**Fig 6:**
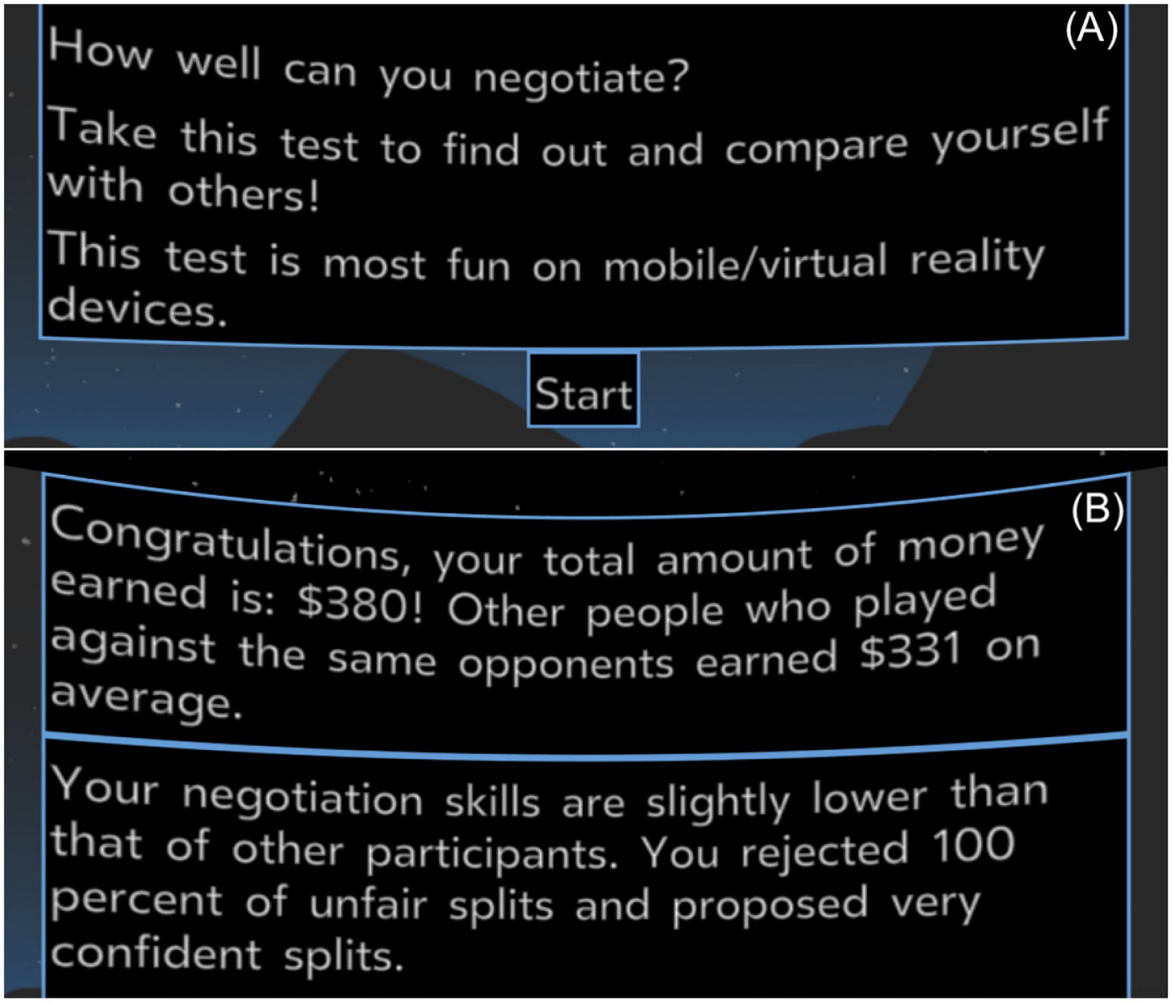
(A) The recruitment page of the negotiation study. Participants are prompted to participate in order to learn about their negotiation skills. (B) The results page of the negotiation study. Participants are assessed based on the total amount of virtual money earned and on their confidence in negotiation.

Our study provides the participants with an assessment of their negotiation skills. We designed the study such that participants get assessed based on their total gain during all negotiations, as well as their confidence in negotiating (what splits were offered and accepted). After all eight negotiation trials, participants received their negotiation assessment based on those two measures. Participants were also provided an explanation of how to interpret their scores. See Fig 6 for screenshots of the recruitment and results pages.

#### Participants

We report on data collected in a time range of two months. During this time, 1334 volunteers (69% male, 31% female) from 57 countries completed the experiment on the platform. Before the start of the test, participants were asked whether they had any previous experience with virtual environments, and how often they generally use computers.

#### Design and analysis

There were three measures of interest: amount offered by participant in the first rounds of each negotiation partner, so trials 1 and 5 (we refer to this as Split 1), amount offered by participant in the third rounds of each negotiation partner, so trials 3 and 7 (we refer to this as Split 2), and whether the participant accepted the unfair split by the confederate in the final rounds with each negotiation partner, so trials 4 and 8 (we refer to this as Unfair Offer).

To analyze Split 1, we ran an analysis of variance (ANOVA) with height as the between-subject factor and Split 1 as the dependent variable. To analyze Split 2, we ran an ANOVA with height as the between-subject factor and Split 2 as the dependent variable. Finally, to test the effect of height on the acceptance rate of the unfair offer, we ran a logistic regression using the acceptance rate as the dependent variable and height as the independent variable.

### Results

The average split offers by participants (65-35 in favor of self), as well as the likelihood to accept unfair splits (22%), were similar to rates reported in prior studies of the Ultimatum Game (60-40, and 22% respectively) [12, 13, 28]. Table 3 shows the mean split behavior by participants’ height conditions.

**Table 3:** Mean and standard deviations (in brackets) of the Split 1 and Split 2 offer type rounds and the unfair offers by the confederate acceptance rate. For comparison, we include the results reported in the original study.

	Our Study		Original Study	
	Short	Tall	Short	Tall
**Split 1** (**$**)	63 (16)	68 (21)	55 (12)	54 (10)
**Split 2** (**$**)	63 (15)	68 (18)	52 (7)	61 (7)
**Unfair Offer Accept**. **Rate** (%)	0.24 (0.43)	0.19 (0.38)	0.72 (0.46)	0.38 (0.50)

The ANOVA to analyze Split 1 did show a statistically significant difference between height conditions *F*(1, 2667) = 6.17, *p* = .0131. We found that participants in the tall condition offered splits significantly more in their favor compared to participants in the short condition. The original study did not find a significant difference in for the Split 1 analysis.

The ANOVA to analyze Split 2 also showed a statistically significant difference between different height conditions *F*(1, 2667) = 6.47, *p* = .0110. This result aligns with the results in the original study, and we observed participants in the tall condition offering splits on average more in their favor compared to participants in the short condition.

The logistic regression to analyze the final, Unfair Offer did not show a statistically significant difference between the tall and short conditions $\chi_{1,N=2668}^{2}=1904,p>0.17$. This stands in contrast with the original study, which found more likely acceptance by participants in the short condition in this analysis.

Finally, we tested the interaction effect between height condition and the device. Adding the device type, and the interaction of device and height, however, did not show a significant interaction effect on all three measures.

To evaluate the effect of uncompensated, unsupervised settings on the data that is collected, we looked at the effect size difference between our data and the data as reported in the original paper for Split 2. We evaluate the effect size with Cohen’s d, and in the original negotiation study, Cohen’s d was 1.23, while in our study, Cohen’s d is 0.34.

### Discussion

In this second study, we showed it is possible to run uncompensated online experiments in virtual environments delivering embodiment illusion. We were able to replicate the results in the original paper at least partially, with some decrease in effect size. We also found no effect of device type on this effect (RQ3), suggesting that embodiment illusion is an equally effective mechanism independent of the device, in this experiment. This suggests that the embodiment illusion is an effective mechanism to investigate peoples’ impact of their appearance on their behavior in uncompensated and unsupervised settings (RQ2).

## Overall discussion

### User devices

One of our research questions asks about the population of online experiments in virtual environments, and what their experiences with different VR devices and HMD was (RQ4).

Table 4 shows the devices that participants used, aggregated over participants of both of our studies. The majority of users seemed to have used Desktop setups. Still about 30% used either mobile or head mounted devices.

**Table 4:** Devices used by participants who completed the tests, aggregated over our two tests.

Device	N
Desktop	1492
Mobile	126
Headmounted	27

Table 5 shows the participants’ experience levels with HMD. A relatively small fraction of people reported significant experience with such devices, and we were surprised how many participants reported to not have any experience using an HMD. This suggests that, while there is a potentially a large number of users, the technology is still in its early stages in terms of adoption at the platform we used.

**Table 5:** Experience with HMD devices of participants on the experiment platform, aggregated over our two test.

HMD Device Usage	N
Never	479
Tried it before	591
Once a week	378
A few times a week	187
At least once a day	10

A large number of our participants were in desktop settings. We were able to replicate most of the findings, however, it remains an open question how much immersion is needed to give a sense of presence in such settings. Previous research suggests that more immersion is better, and we believe it is a tradeoff to build VR experiments with more immersion, while still laying keeping the experience accessible. The difference in effect sizes between the original, lab-based studies and ours showed that our experiments led to smaller effect sizes. We suspect that this difference comes from less immersion and the simplified experiment in our study as compared to the original experiment. However, our larger number of participants means that these effects are still robustly detectable. We found that device effects on results were only secondary and did not interact with any of the main results, and devices only affected error rates in the maze study.

### Demographics

**Fig 7:**
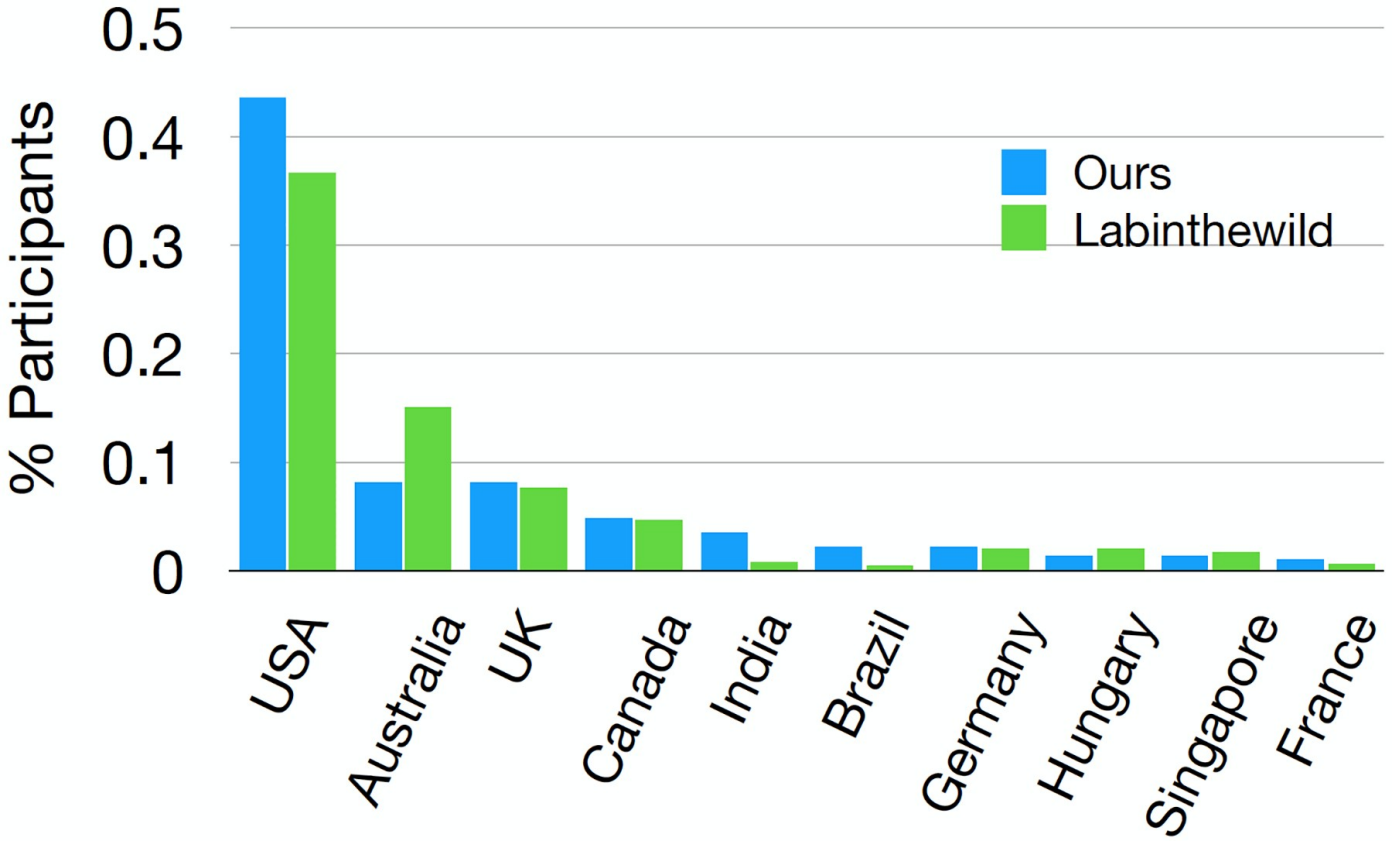
Nationality distribution of both of our experiments, or participants who completed our tests. We compare our demographics distribution with the data reported from LabintheWild as reported in [3].

One of the previously reported advantages of running experiments online in uncompensated settings is the potential diversity of the participant pool. We therefore also looked at the demographics distribution of participants of the two studies (RQ4). Table 6 provides an overview of age and gender distribution of our participants vs. participants on LabintheWild [3]. Fig 7 shows nationality distribution of our participants, also as compared to the distribution of participants on LabintheWild [3]. Participants in our studies came from over 60 different nationalities, with the chart showing the ten most popular nationalities.

**Table 6:** Age and gender distribution of both of our experiments of participants who completed our tests.

Platform	Age (years)	Gender (%)
Ours	Mean: 28.1 Stdev: 11.6	M: 60.5 F: 39.5
LabintheWild	Mean: 29 Stdev: 1.1	M: 51.0 F: 49.0

### Unsupervised experiments in virtual environments

One of the research questions we posed was whether online experiments in virtual environments are feasible in uncompensated settings. We redesigned experiments such that they fit the incentive structures of voluntary participants, such that they receive feedback and could learn about themselves. While our studies showed reasonable traffic, we believe that designing the incentive structures is even more crucial in such experiments. We believe that the design of intrinsically motivating experiments is, therefore, a promising research avenue to be further explored.

One of the common objections of running studies online is how data quality is affected. In our second study, we compared the effect sizes of an online study with a lab-study. Our analysis suggests that effect sizes get lower, which could be due to the lack of control over subjects and device use.

It remains an open question of what controllers and interactions people at uncompensated experiment platforms prefer that also leads to the best user experience. Since there is a lot of research going into how users can interact in virtual environments, it becomes necessary to investigate how to transfer such interactions to unsupervised online settings.

## Limitations

One of the limitations of our work is that the sample sizes are relatively small compared to other online studies. We were still able to replicate most of the results, while larger-scale studies will give a better view of demographics. We believe that VR is still in very early stages, and adoption will bring more momentum to participants of such tests.

Another limitation of our work is that the sample population seems still biased towards male participants. Given that in other platforms of similar kind, the population has been shown to be more diverse, one factor could be that our current design did not appeal to all participants equally. In future studies, we will focus more on designing the study to be appealing to a more diverse group of users. Diversity in the user population of VR can be for example achieved with testing different recruitment strategies or may be achieved through further technology adoption.

## Conclusion

We studied uncompensated and unsupervised samples, in which researchers have little control over devices people use to access the site. These are important and known challenges with VR experiments [22], underlining the importance to validate whether VR experiments are feasible to be studied in such settings. We studied two phenomena in virtual environments: In a navigation study to identify gender differences in a spatial navigation task in a virtual environment, we analyze gender differences in a navigation task in virtual mazes. In a negotiation study to investigate how participant appearance affects confidence, participants heights were manipulated with taller participants revealing more confident negotiation behavior. We reproduce the experiments and find that results hold across device types.

Our data demonstrate that we were able to replicate key results from the original studies, but the effect sizes in our replication of Study 2 were smaller than in the original paper. These are important findings for anyone seeking to conduct VR studies with uncompensated online samples because they demonstrate both the feasibility of the research and the need to allow for larger sample sizes (which, fortunately, online experimentation makes easy). Finally, as technology adoption occurs, we will be able to more accurately investigate device differences.
